# LINC00114 stimulates growth and glycolysis of esophageal cancer cells by recruiting EZH2 to enhance H3K27me3 of DLC1

**DOI:** 10.1186/s13148-022-01258-y

**Published:** 2022-04-12

**Authors:** Jianzhang Qin, Yishuai Li, Zhe Li, Xuebo Qin, Xuetao Zhou, Hao Zhang, Shujun Li

**Affiliations:** 1 Department of Hepatobiliary Surgery, The Second Hospital of Hebei Medical University, Shijiazhuang, Hebei China; 2 Department of Thoracic Surgery, Hebei Chest Hospital, Shijiazhuang, Hebei China; 3 Department of Thoracic Surgery, Shijiazhuang People’s Hospital, Shijiazhuang, Hebei China; 4 Department of Cardiothoracic Surgery, Shijiazhuang Third Hospital, Shijiazhuang, Hebei China; 5 Department of Pathology, Institute of Precision Cancer Medicine and Pathology, Jinan University Medical College, Guangzhou, Guangdong China; 6 Department of Thoracic Surgery, The Second Hospital of Hebei Medical University, 215 West Heping Road, Shijiazhuang, Hebei China

**Keywords:** Esophageal cancer, LINC00114, Enhancer of zeste homolog 2, Deleted in liver cancer 1, H3K27me3, Glycolysis

## Abstract

**Objective:**

LINC00114 could promote the development of colorectal cancer, but its mechanism has been rarely discussed in esophageal cancer (EC). Herein, we explored the molecular mechanism of LINC00114 via mediating enhancer of zeste homolog 2/deleted in liver cancer 1 (EZH2/DLC1) axis in EC.

**Methods:**

LINC00114, EZH2 and DLC1 expression in EC tissues and cells were tested. LINC00114, EZH2 and DLC1 expression were altered in EC cells through transfection with different constructs, and cell proliferation, migration, invasion, apoptosis and glycolysis were subsequently observed. The interaction between LINC00114 and EZH2 and that between EZH2 and DLC1 were explored. Tumor formation was also conducted to confirm the in vitro results.

**Results:**

The expression levels of LINC00114 and EZH2 were elevated while those of DLC1 were reduced in EC. Inhibiting LINC00114 or reducing EZH2 blocked cell proliferation, migration, invasion and glycolysis and induce cell apoptosis in EC. LINC00114 promoted H3K27 trimethylation of DLC1 by recruiting EZH2. Knockdown of DLC1 stimulated cell growth and glycolysis in EC and even mitigated the role of LINC00114 inhibition in EC. In vivo experiment further confirmed the anti-tumor effect of LINC00114 inhibition in EC.

**Conclusion:**

The data indicate that LINC00114 promotes the development of EC by recruiting EZH2 to enhance H3K27me3 of DLC1.

**Supplementary Information:**

The online version contains supplementary material available at 10.1186/s13148-022-01258-y.

## Introduction

Esophageal cancer (EC) refers to a malignancy in the esophagus, and it has an unfavorable prognosis [1]. EC mainly includes two entities esophageal squamous cell carcinoma (ESCC) and esophageal adenocarcinoma that have distinct epidemiological and pathological characteristics in anatomy [2]. Except for a symptom in the early stage, patients with ESCC complain dysphagia, back pressure, unconscious weight loss, epigastric pain, discomfort, or burning sensation [3]. In the elderly, endoscopic or surgery is preferred to the disease in the early stage while surgery with chemotherapy for the advanced disease [4]. Tumor occurrence depends on the reprogramming of cell metabolism. The changes in intracellular and extracellular metabolites accompanying cancer-related metabolic reprogramming have profound effects on gene expression, cell differentiation, and tumor microenvironment [5, 6]. Long non-coding RNA (LncRNA) could mediate histone modification and DNA methylation in EC [7] and regulate cancer markers, such as tumor growth and metabolism [8]. Given that, targeting lncRNA may be a promising method to control the disease development.

In EC, various lncRNAs have been suggested to regulate cellular biology. It is recently known that suppression of lncRNA gradually increased during hepatocarcinogenesis delays the progression of EC via the ceRNA network [9], and the same regulatory mechanism has been observed in small nucleolar RNA host gene 1-regulated EC progression [10]. Long non-coding RNA 00114 (LINC00114) has been indicated to stimulate the progression and radioresistance of nasopharyngeal carcinoma (NPC) [11]. Also, a study has shown that LINC00114 serves as an oncogenic role in colorectal cancer (CRC) via regulating enhancer of zeste homolog 2 (EZH2)-mediated methylation of the target gene [12]. However, the mechanism of LINC00114 in EC remains elusive. EZH2 is a vital mediator of gene expression by H3K27me3 that emerges as a novel agent in tumor treatment [13]. In fact, overexpression of EZH2 has been reported in EC [14] and is related to the dismal overall survival of patients with EC [15]. Notably, EZH2 recruitment is a key in a lncRNAs-mediated network in EC [16, 17] and up-regulation of EZH2 and H3K27me3 is indicative of an inferior prognosis of ESCC patients [18]. Mechanistically, EZH2 reduction could reduce the enrichment of H3K27me3 in the promoter of deleted in liver cancer 1 (DLC1), thereby inhibiting tumorigenesis and metastasis [19]. DLC1 is often silenced in human cancers [20] and is promising to suppress tumor development in ESCC [21]. This research was initiated with the speculation that LINC00114 stimulated the progression of EC through recruiting EZH2 to enhance H3K27me3 of DLC1.

## Methods and materials

### Ethics statement

All subjects signed an informed consent form. Specimen collection was approved by the Ethics Committee of the Second Hospital of Hebei Medical University (ethical committee approval code: 20190106). Animal treatments were performed following the Guideline of Experimental Animal Ethics Committee of the Second Hospital of Hebei Medical University (ethical committee approval code: 20190614).

### Clinical samples

The 89 surgically resected specimens (63 males and 26 females, 25–71 years old, with an average age of 50 years) admitted to the Second Hospital of Hebei Medical University were confirmed as EC by the pathological examination. The non-tumoral tissues (3–5 cm from the tumor) were collected. None of the patients received anti-cancer treatment prior to our analysis. The tissues were stored at − 80 °C [16].

### Cell culture

Human esophageal epithelial cells (HEEC) and EC cell lines (Eca109, TE1, KYSE150 and KYSE450) were provided by the BeNa Culture Collection (Beijing, China). The EC cell line was maintained in Roswell Park Memorial Institute (RPMI)-1640 medium supplemented with 10% fetal bovine serum (FBS; #04-001-1ACS, BI), 100 U/mL penicillin and 100 μg/mL streptomycin under 37 °C and 5% CO_2_ [22].

### Cell transfection

EC cells seeded in 6-well plates at a density of 2 × 10^6^ cells/well were incubated until the cells reached 50% confluence. Lipofectamine 2000 (Invitrogen, CA, USA) was used for EC cell transfection according to manufacturer’s instructions [23].

EC cells were transfected with shRNA NC, sh-LINC00114-1&2, shEZH2-1&2, si-DLC1-1&2 or co-transfected with sh-LINC00114 + si-NC, or sh-LINC00114 + si-DLC1. Various constructs were synthesized from Genechem (Shanghai, China) and Generay Biotech (Shanghai, China). The transfection efficiency was determined by reverse transcription quantitative polymerase chain reaction (RT-qPCR) and Western blot assay.

### RT-qPCR

After being extracted from tissues and cells using Trizol (Invitrogen), RNA was quantified using Nanodrop2000 (1011U, NanoDrop Technologies, DE, USA). Reverse transcription was conducted to generate cDNA using iSCRIPT cDNA Synthesis Kit (Bio-Rad, Glattbrugg, Switzerland). qRT-PCR were prepared using SYBR Green kit (Qiagen). The primers were compounded by Gene Pharma (Shanghai, China) (Additional file 1: Table S1) and glyceraldehyde-3-phosphate dehydrogenase (GAPDH) was the internal control. On the ABI 7500 quantitative PCR instrument (7500, ABI, NY, USA), PCR was performed. Data quantification was conducted via 2^−ΔΔCT^ method [24].

### Western blot assay

Total proteins were extracted using Radio-immunoprecipitation assay (RIPA) lysis buffer containing phenylmethanesulfonyl fluoride (PMSF; Solarbio, Beijing, China). Protein concentration was measured using bicinchoninic acid kit (Yeasen, Shanghai, China). After sodium dodecyl sulfate polyacrylamide gel electrophoresis, the protein was transferred to the polyvinylidene fluoride membrane, blocked with 5% skimmed milk powder and added with primary antibodies DLC1 (1:100, Santa Cruz Biotechnology), EZH2 (1:1000) and GAPDH (1:2500, both from Abcam). Subsequently, the membrane was incubated with HRP-labeled secondary antibody (1:2000, Abcam) and visualized by the enhanced chemiluminescence kit (Ameshame, UK). The images were captured by the system (Bio-Rad, CA, USA) and data were analyzed by Quantum One v4.6.2 software.

### Cell glycolysis

Glucose uptake was determined using Glucose Uptake Colorimetric Assay Kit (Abcam, Cambridge, MA, USA) according to the manufacturer’s instruction. Lactate Assay Kit II and ATP Colorimetric Assay Kit (Biovision, Mountain View CA, USA) were used to measure production of lactate and ATP according to the manufacturer’s instructions. Measurements were performed at least three replicates and then averaged [25, 26].

### Colony formation assay

EC cells were added to 6-well plates at a density of 1000 cells/well and incubated in an incubator containing 5% CO_2_ at 37 °C. After 14 d, colonies were fixed with paraformaldehyde for 15 min and stained with 0.1% crystal violet solution for 15 min. The colonies containing more than 50 cells were manually counted using ImageJ. Data were normalized to the control group.

### Cell counting kit (CCK)-8 assay

CCK-8 kit (GLPBIO, Shanghai, China) was used for assessing cell proliferation. Cells were plated into 96-well plate at a density of 3000 cells/well, added with CCK-8 solution (100 µL/well) and cultured for 2 h. Optical density value was measured using a microplate reader (BioTek Instruments) at 450 nm [17, 27].

### RNA immunoprecipitation (RIP)

According to the protocol of manufacturer, the RIP kit (Millipore, MA, USA) was used to measure the binding of LINC00114 to EZH2. Cells were cultured in the 6-well plate to reach 80–90% confluence, lysed in an ice bath with an equal volume of RIPA lysis buffer (Beyotime, Shanghai, China) for 5 min, and centrifuged at 14,000 rpm for 10 min at 4 °C. A part of the supernatant was co-precipitated by incubating with the antibody EZH2 (1:100, Abcam), H3K27me3 (1:100, Abcam) or IgG (1:100, Abcam) for 30 min. Briefly, 50 µL magnetic beads from each co-precipitation reaction system were resuspended in 100 µL RIP wash buffer and incubated with 5 µg corresponding antibody. Subsequently, the magnetic bead-antibody complexes were resuspended in 900 µL RIP wash buffer and, then incubated with 100 µL cell lysate overnight at 4 °C, and the magnetic beads-protein complex was collected. Afterward, samples were treated with proteinase K to extract RNA for subsequent PCR detection of LINC00114 [17].

### RNA-pull down assay

Cells were transfected with biotinylated LINC00114 and LINC00114 antisense RNA (50 nM each). At 48 h post-transfection, the cells were lysed in the lysis buffer (Ambion, TX, USA) for 10 min. Taking 50 mL cell lysate as a control, the remaining lysate was incubated with M-280 streptavidin magnetic beads pre-coated with RNase-free and yeast tRNA (Sigma, USA) for 3 h at 4 °C and washed twice with cold lysis buffer, 3 times with low-salt buffer, and once with high-salt buffer. After that, the total protein was extracted with RIPA lysis buffer and incubated with EZH2 antibody (1:100, Abcam) for Western blot analysis [17].

### Chromatin immunoprecipitation (ChIP)

ChIP analysis was performed referring the instructions of EZ Chip Kit (Millipore). The antibodies H3K27me3 (Abcam) and EZH2 (Abcam) and rabbit IgG (Abcam) were utilized. DLC1 primers: 5-CCACCTCCGCCAAGTAAATGC-3′ (forward) and 5′-CCGAAAAGTCGCCAACTATTG-3′ (reverse). ABI Prism 7500 (Applied Biosystems) was utilized for quantifying gene expression [19].

### Transwell assay

Transwell chamber was used for determining migration and invasion of EC cells. Migration test: cells were starved for 24 h (cultured in FBS-free RPMI-1640 medium) and suspended in FBS-free RPMI-1640 medium to prepare cell suspension at a density of 2 × 10^5^ cells/mL after detachment and centrifugation. About 0.2 mL suspension was added to the upper chamber and 700 μL RPMI-1640 medium containing 10% FBS to the lower chamber.

Invasion test: Matrigel (Corning) was diluted with serum-free medium at 1:9 to a final concentration of 1 mg/mL, coated on the 24-well Transwell upper chamber with a total volume of 40 μL, and incubated for 5 h. In the upper chamber, 70 μL RPMI-1640 medium was added while in the lower chamber, 700 μL RPMI-1640 medium containing 10% FBS was supplemented. Cells were starved for 24 h by reducing the serum concentration in the culture medium and resuspended in FBS-free RPMI-1640 medium to maintain a density of 2.5 × 10^5^ cells/mL, and 0.2 mL suspension was incubated in the upper chamber.

Incubated for 24 h, cells that migrated or invaded were fixed with methanol for 30 min and dyed with 0.1% crystal violet solution for 20 min. Under an inverted microscope, five fields were selected to count the number of cells that passed through the membrane.

### Flow cytometry

Cells were detached with 0.25% ethylene diamine tetraacetic acid-depleted trypsin and collected after centrifugation. Cells were resuspended in 1 × binding buffer and detected by Annexin V-fluorescein isothiocyanate (FITC)/propidium iodide (PI) kit (BD Pharmingen, San Diego, USA) following the manufacturer's instructions. Apoptotic cells were tested by a flow cytometer (BD Biosciences, NJ, USA), and the apoptosis rate was then calculated [26].

### Tumor xenografts in nude mice

Eca109 cells (0.2 mL, 5 × 10^6^ cells) that stably transfected with sh-LINC00114 or si-DLC1 were injected subcutaneously into female BALB/c nude mice (4 weeks old). The mice were supplied by Beijing Vital River Laboratory Animal Technology Co., Ltd. (Beijing, China). Basic condition and individual mouse weight were monitored during the whole period. Tumor volume was determined every 5 days by calliper measurements. Tumor volume = 0.5 × Length × Width^2^. After 4 w, mice were euthanized, and the average tumor weight was measured [28].

### Immunohistochemistry

Paraffin-embedded sections were deparaffinized, blocked with goat serum blocking solution (Shanghai Haoran Biological Technology Co., Ltd., Shanghai, China) and incubated with primary antibody DLC1 (1:250, Santa Cruz Biotechnology, USA) overnight at 4 °C. Afterward, the sections were added with secondary antibody for 20 min, incubated with horseradish peroxidase (HRP)-labeled streptomyces ovalbumin (Beijing Yi Mao Biotechnology Co., Ltd.), and developed by diaminobenzidine (WI-IIGA, Guangzhou, China). Then, the sections were treated with hematoxylin (Shanghai Bogoo Biotechnology Co., Ltd., Shanghai, China), rinsed with 1% ammonia, dehydrated with gradient alcohol, cleared with xylene and mounted with neutral resin. The final result was evaluated by double-blind method. Five high-magnificence fields were randomly selected under an optical microscope (Olympus, Japan) to observe positive cells. The percentage of positive cells in total cells was calculated.

### Statistical analysis

Statistical analysis was implemented using SPSS 21.0 (IBM, NY, USA). All data were normally distributed and in homogeneity of variance. Measurement data were presented as mean ± standard deviation. Paired *t* test was used for comparison between cancer tissues and non-tumoral tissues, while an independent sample t test for that between the other two groups. One-way analysis of variance (ANOVA) was applied for comparison among multiple groups, and Tukey method for post-hoc test. *P* < 0.05 suggested statistical difference.

## Results

### LINC00114 expression level is raised in EC; suppression of LINC00114 inhibits growth and glycolysis of EC cells

**Fig. 1:**
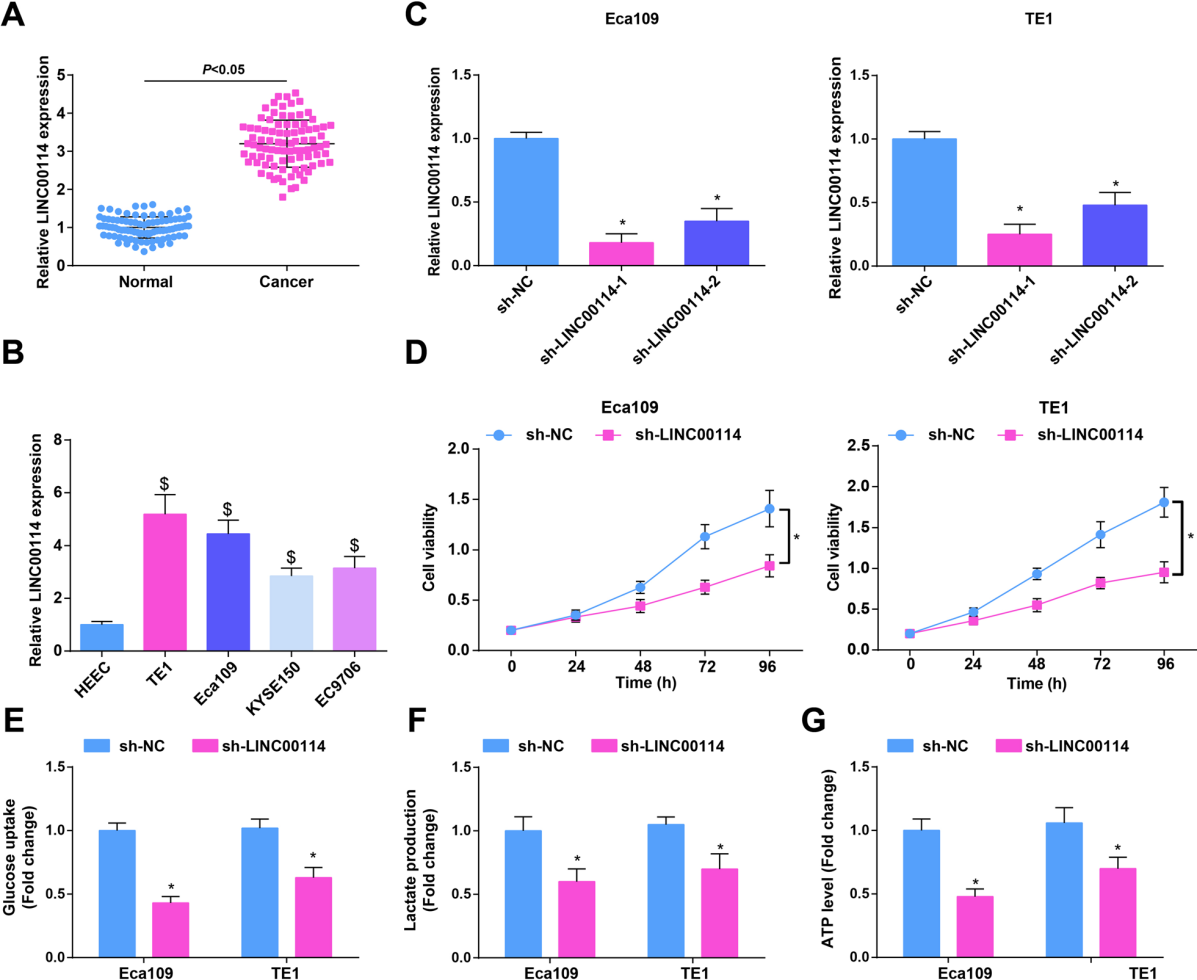
LINC00114 expression level is raised in EC; Suppression of LINC00114 blunts growth and glycolysis of EC cells. **A** RT-qPCR detected LINC00114 expression in EC and non-tumoral tissues (*n* = 89); **B** RT-qPCR detected LINC00114 expression in EC cells; **C** RT-qPCR detected the transfection efficiency of sh-LINC00114 in EC cells; **D** CCK-8 detected proliferation of EC cells after LINC00114 interference; **E** colorimetry detected glucose consumption in EC cells after LINC00114 interference; **F** lactic acid determination kit II detected lactic acid production in EC cells after LINC00114 interference; **G** ATP colorimetric determination kit detected ATP level in EC cells after LINC00114 interference; Data were shown as mean ± standard deviation. *N* = 3, $ *P* < 0.05 versus HEEC; *P < 0.05 versus the sh-NC group

Reports have indicated that LINC00114 is up-regulated in CRC and NPC [11, 12]. To explore the possible role of LINC00114 in EC, LINC00114 expression in 89 pairs of EC tissues and non-tumoral tissues was measured by RT-qPCR, and the data revealed that LINC00114 expression levels were higher in EC tissues compared to non-tumoral tissues (Fig. 1A). The same trend was also tested in human EC cell lines compared with HEEC cell line, among which LINC00114 expression in Eca109 cells and TE1 cells was higher than other EC cell lines (Fig. 1B). Therefore, Eca109 and TE1 cells were utilized for further cellular experiments.

Two shRNAs targeting LINC00114 were designed to inhibit LINC00114 expression in cells. After transfection, RT-qPCR results suggested that both shRNAs caused the decrease in LINC00114 expression, and the more effective shRNA (sh-LINC00114-1) was selected for subsequent experiments (Fig. 1C). CCK-8, colony formation and Transwell assays, along with flow cytometry were adopted for testing cell proliferation, migration, invasion and apoptosis. The findings displayed that after LINC00114 down-regulation, proliferation, migration and invasion of Eca109 and TE1 cells were reduced while cell apoptosis was promoted (Fig. 1D; Additional file 2: Fig. S1A–D).

Malignant cells present increased glycolysis and rely on the production of ATP to promote active metabolism and proliferation [29]. Thus, the effects of LINC00114 on glucose uptake, lactate production and ATP were explored, and the outcomes depicted that after down-regulating LINC00114, glucose uptake, lactate production and ATP levels in Eca109 cells and TE1 cells were reduced (Fig. 1E–G).

Our results concluded that inhibiting LINC00114 suppressed the growth and glycolysis of EC cells.

### LINC00114 binds with EZH2; silencing EZH2 inhibits the growth and glycolysis of EC cells

**Fig. 2:**
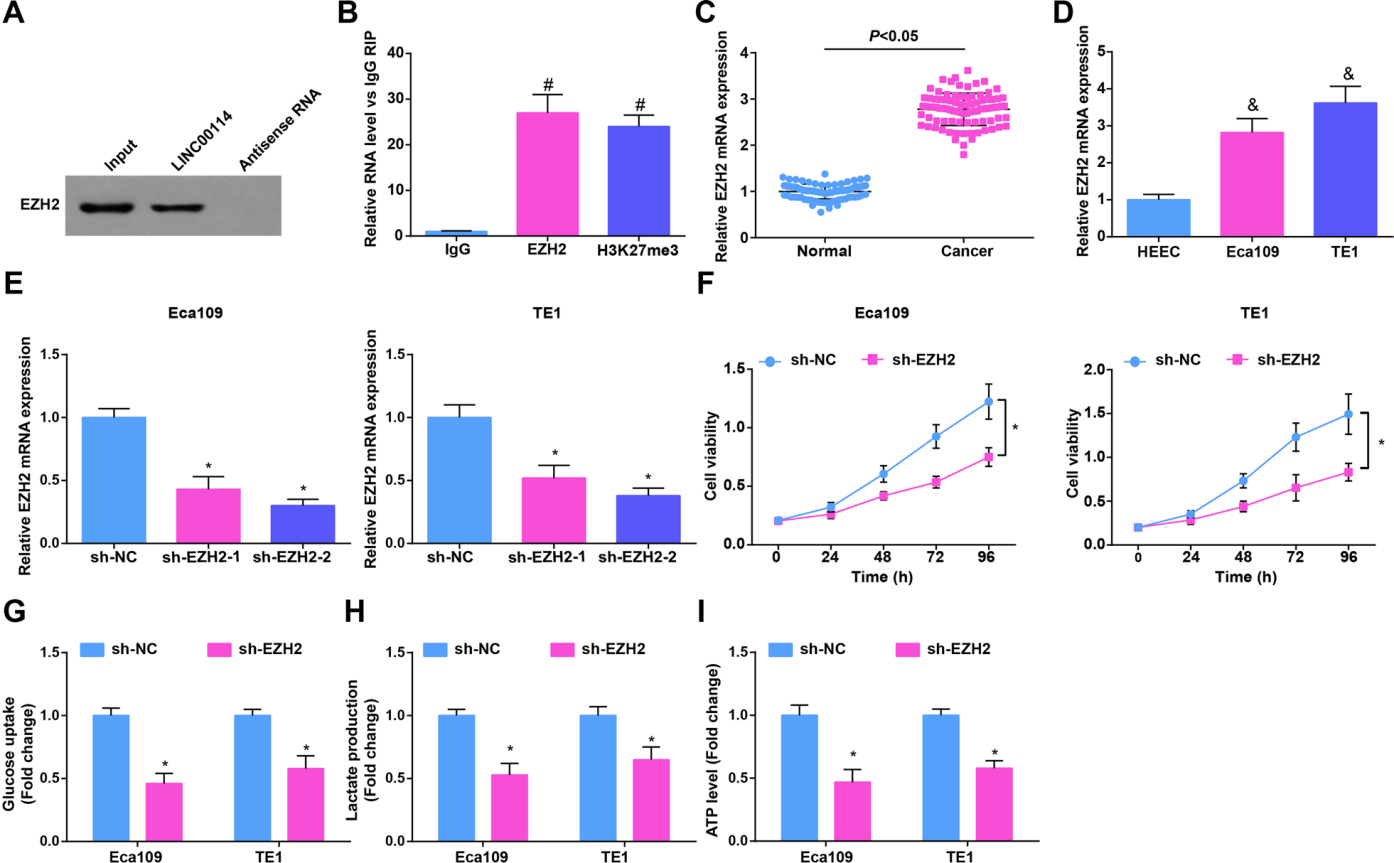
LINC00114 binds with EZH2; silencing EZH2 inhibits growth and glycolysis of EC cells. **A** RNA pull-down experiment observed whether LINC00114 can bind to EZH2; **B** RIP assay detected the binding between LINC00114 and EZH2; **C**, **D** RT-qPCR detected EZH2 expression in tissue samples (EC tissues and non-tumoral tissues) and cells (HEEC and EC cells); **E** RT-qPCR detected the transfection efficiency of sh-EZH2 in EC cells; **F** CCK-8 detected proliferation of EC cells after EZH2 interference; **G** colorimetry detected glucose consumption in EC cells after EZH2 interference; **H** lactic acid determination kit II detected lactic acid production in EC cells after EZH2 interference; **I** ATP colorimetric determination kit detected ATP level in EC cells after EZH2 interference; Data were shown as mean ± standard deviation. *N* = 3; # *P* < 0.05 versus IgG. & *P* < 0.05 versus HEEC. *P < 0.05 versus the sh-NC group

It is previously reported that lncRNA inhibits E-cadherin transcription by binding to EZH2 in liver cancer cells [30]. To explore the interaction between LINC00114 and EZH2, RNA pull-down assay was conducted, and the finding implied that LINC00114 could bind to EZH2 (Fig. 2A). Based on the evidence that trimethylation of H3K27 causes gene silencing and transcriptional suppression [31], RIP assay was performed (Fig. 2B), and the outcome suggested that LINC00114 was enriched in the immunoprecipitates under EZH2 and H3K27me3 antibody treatment. The above results indicated that LINC00114 could promote the trimethylation of H3K27 by recruiting EZH2.

Next, EZH2 expression in EC was examined by RT-qPCR, presenting an increase in EC tissues and cells (Fig. 2C, D). Transfection of sh-EZH2 was carried out in EC cells, and RT-qPCR showed that the transfection efficiency of sh-EZH2-2 was better (Fig. 2E). Subsequently, experimental data analyzed that silencing EZH2 suppressed proliferation, migration and invasion ability and enhanced apoptosis (Fig. 2F; Additional file 3: Fig. S2A–D), as well as reduced glucose uptake, lactate production and ATP levels in Eca109 and TE1 cells (Fig. 2G–I). The results show that interference with EZH2 inhibited the proliferation, migration and glycolysis of EC cells.

### LINC00114 recruits EZH2 to increase the enrichment of H3K27me3 in the DLC1 promoter

**Fig. 3:**
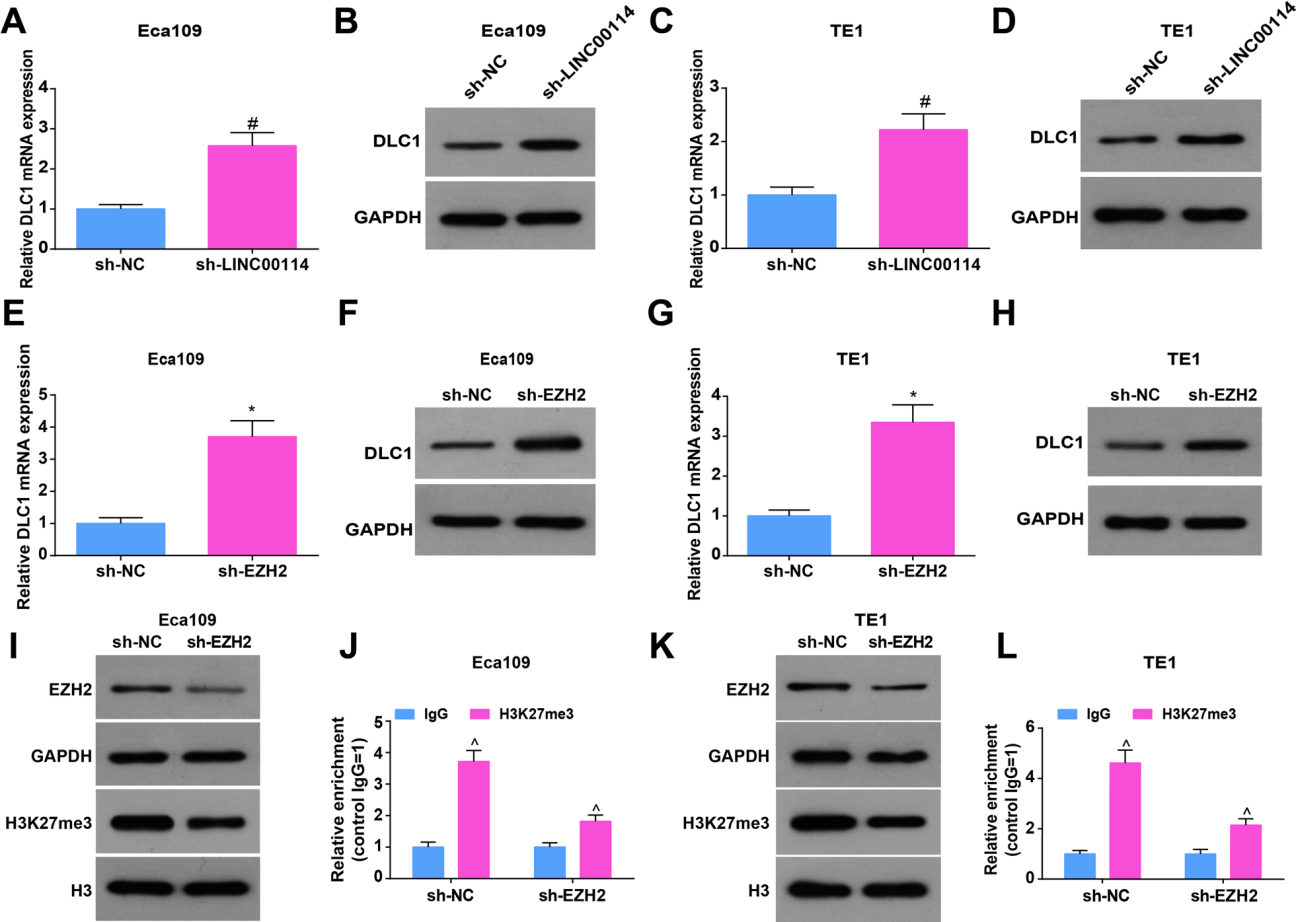
LINC00114 recruits EZH2 to increase the enrichment of H3K27me3 in the DLC1 promoter. **A**, **B** RT-qPCR and Western blot detected the mRNA and protein expression levels of DLC1 in Eca109 cells after interfering with LINC00114; **C**, **D** RT-qPCR and Western blot detected the mRNA and protein expression levels of DLC1 in TE1 cells after interfering with LINC00114; **E**, **F** RT-qPCR and Western blot detected the mRNA and protein expression levels of DLC1 after interference with EZH2 in Eca109 cells; **G**, **H** RT-qPCR and Western blot detected the mRNA and protein expression levels of DLC1 after interference with EZH2 in TE1 cells; **I** Western blot detected EZH2 and H3K27 protein levels after interference with EZH2 in Eca109 cells; **J** ChIP experiment detected H3K27me3 enrichment of DLC1 promoter in Eca109 cells after interference with EZH2; **K** Western blot detected EZH2 and H3K27 protein levels in TE1 cells after interference with EZH2; **L** ChIP experiment detected H3K27me3 enrichment of DLC1 promoter after interference with EZH2 in TE1 cells; Data were shown as mean ± standard deviation. *N* = 3, in **A**, **B**, **E**, **F**, **I**, # *P* < 0.05 versus the sh-NC group; in **C**, **D**, **G**, **H**, **K**, *P < 0.05 versus the sh-NC group. ^ *P* < 0.05 versus IgG

H3K27me3 is enriched in the DLC1 promoter of MHCC97L cells that do not express DLC1 [19] and DLC1 is frequently silenced by methylation [32]. To further explore the mechanism among LINC00114, EZH2 and DLC1, RT-qPCR and Western blot assays were conducted, finding that down-regulating LINC00114 in Eca109 and TE1 cells increased DLC1 expression (Fig. 3A–D) while silencing EZH2 elevated DLC1 expression (Fig. 3E–H) and inhibited H3K27me3 expression (Fig. 3I, J). Furthermore, ChIP analysis reflected that H3K27me3 was enriched in the DLC1 promoter, while knocking down EZH2 reduced the enrichment of H3K27me3 in the DLC1 promoter (Fig. 3K, L). The findings all suggested that LINC00114 recruited EZH2 to increase the enrichment of H3K27me3 in the DLC1 promoter.

### Knocking down DLC1 enhances growth and glycolysis of EC cells

**Fig. 4:**
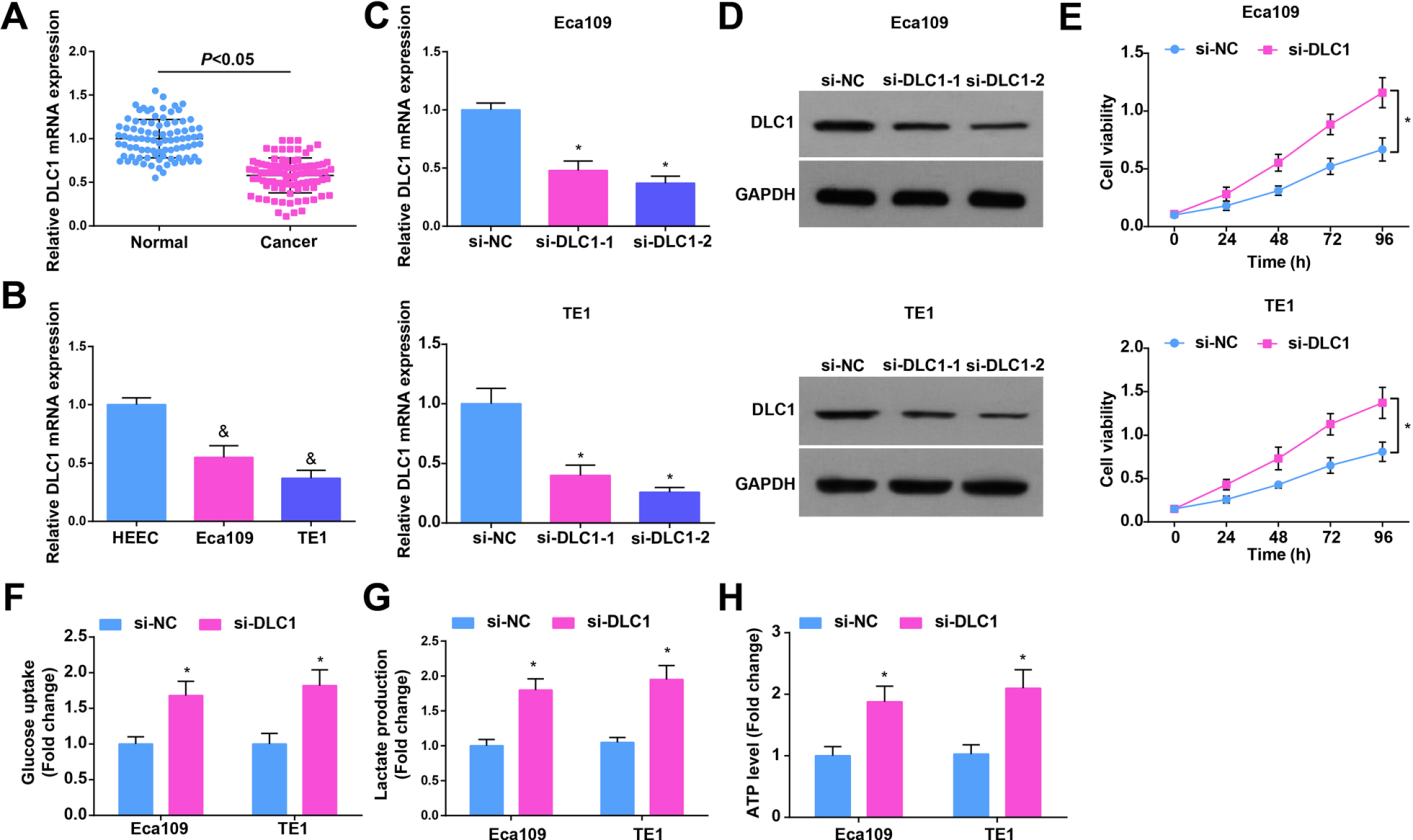
Knocking down DLC1 enhances growth and glycolysis of EC cells. **A**, **B** RT-qPCR detected DLC1 expression in tissue samples (EC tissues and non-tumoral tissues) and cells (HEEC and EC cells); **C**, **D** RT-qPCR and Western blot detected the transfection efficiency of si-DLC1 in Eca109 and TE1 cells; **E** CCK-8 detected proliferation of EC cells after DLC1 interference; **F** colorimetry detected glucose consumption in EC cells after DLC1 interference; **G** lactic acid determination kit II detected lactic acid production in EC cells after DLC1 interference; **H** ATP colorimetric determination kit detected ATP level in EC cells after DLC1 interference; data were shown as mean ± standard deviation. *N* = 3; & *P* < 0.05 versus HEEC. *P < 0.05 versus the si-NC group

DLC1 is defined as a tumor suppressor [33]. To test the role of DLC1 in EC, DLC1 expression in EC was measured by RT-qPCR, showing a low expression level in cancer tissues and cells (Fig. 4A, B). si-DLC1 was transfected into Eca109 and TE1 cells, and si-DLC1-2 was better to down-regulate DLC1 expression (Fig. 4C, D). After DLC1 down-regulation, it was found that cell proliferation (Fig. 4E) was induced, apoptosis (Additional file 4: Fig. S3D) was suppressed and glucose uptake, lactate production and ATP levels were increased (Fig. 4F–H) in Eca109 and TE1 cells. Meanwhile, Eca109 and TE1 cells interfered with DLC1 showed promoted colony formation, invasion and migration abilities (Additional file 4: Fig. S3A–C). Shortly, interference with DLC1 promoted the proliferation, migration, invasion and glycolysis of EC cells and inhibited cell apoptosis.

### Knocked down DLC1 mitigates the role of suppressed LINC00114 in growth and glycolysis of EC cells

**Fig. 5:**
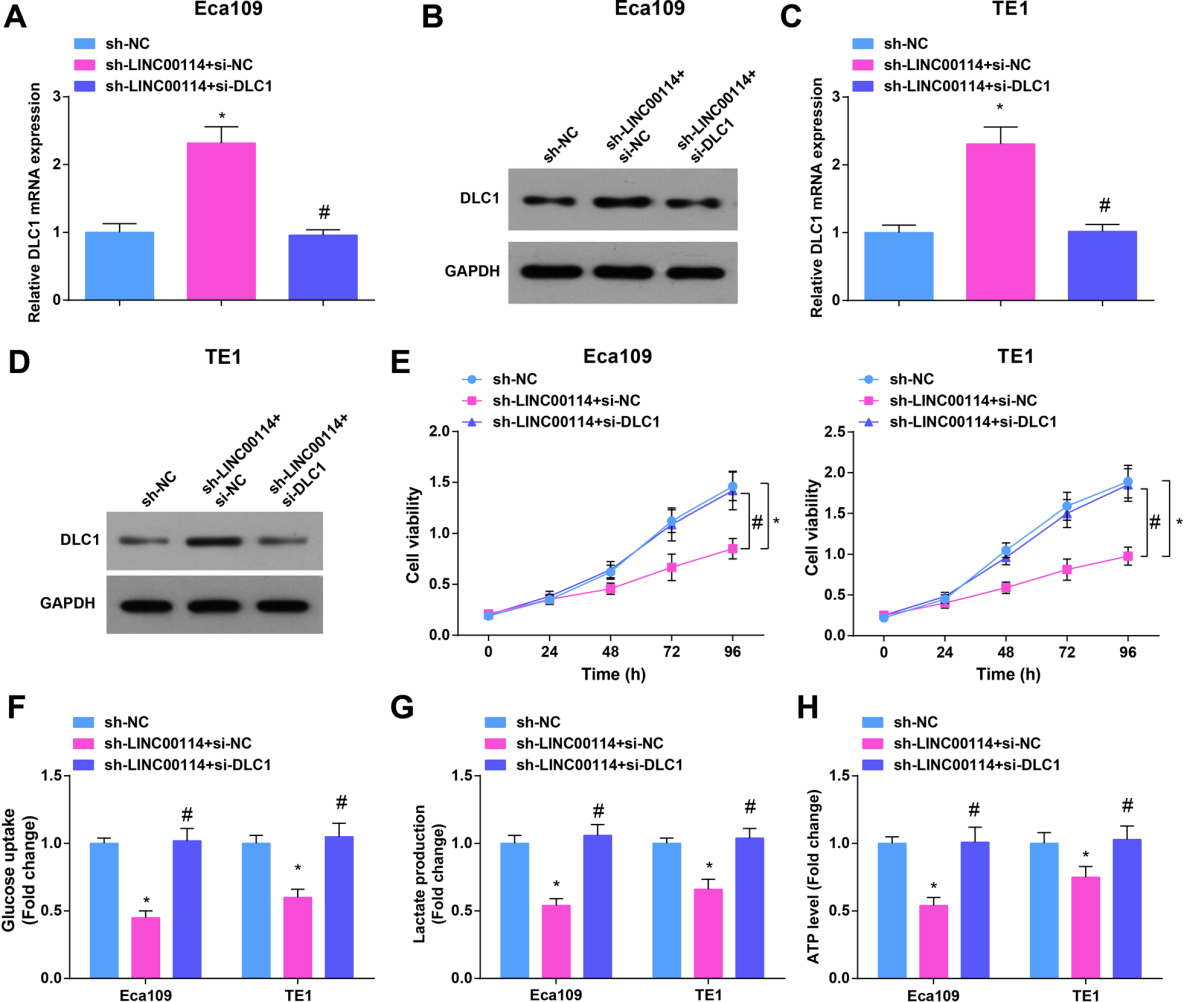
Knocked down DLC1 mitigates the role of suppressed LINC00114 in growth and glycolysis of EC cells. **A**, **B** RT-qPCR and Western blot detected DLC1 expression in Eca109 cells after co-transfection with sh-LINC00114 and si-DLC1; **C**, **D** RT-qPCR and Western blot detected DLC1 expression in TE1 cells after co-transfection with sh-LINC00114 and si-DLC1; **E** CCK-8 detected proliferation of EC cells after co-transfection with sh-LINC00114 and si-DLC1; **F** colorimetry detected glucose consumption in EC cells after co-transfection with sh-LINC00114 and si-DLC1; **G** lactic acid determination kit II detected lactic acid production in EC cells after co-transfection with sh-LINC00114 and si-DLC1; **H** ATP colorimetric determination kit detected ATP level in EC cells after co-transfection with sh-LINC00114 and si-DLC1; data were shown as mean ± standard deviation. *N* = 3; *P < 0.05 versus the sh-NC group. # *P* < 0.05 versus the sh-LINC00114 + si-NC group

To further observe that LINC00114 regulates the biological functions of EC cells through DCL1, we interfered with LINC00114 and DLC1 expression in Eca109 and TE1 cells. It was found that sh-LINC00114-mediated EC cell proliferation and glycolysis were all reversed by si-DLC1 (Fig. 5A–H), as well as cell colony-forming, invasion, migration and apoptosis (Additional file 5: Fig. S4A, B and Additional file 6: Fig. S5A, B).

Shortly, knocking down DLC1 mitigated the role of suppressed LINC00114 in growth and glycolysis of EC cells.

### Down-regulating LINC00114 elevates DLC1 expression to suppress EC tumor growth in vivo

**Fig. 6:**
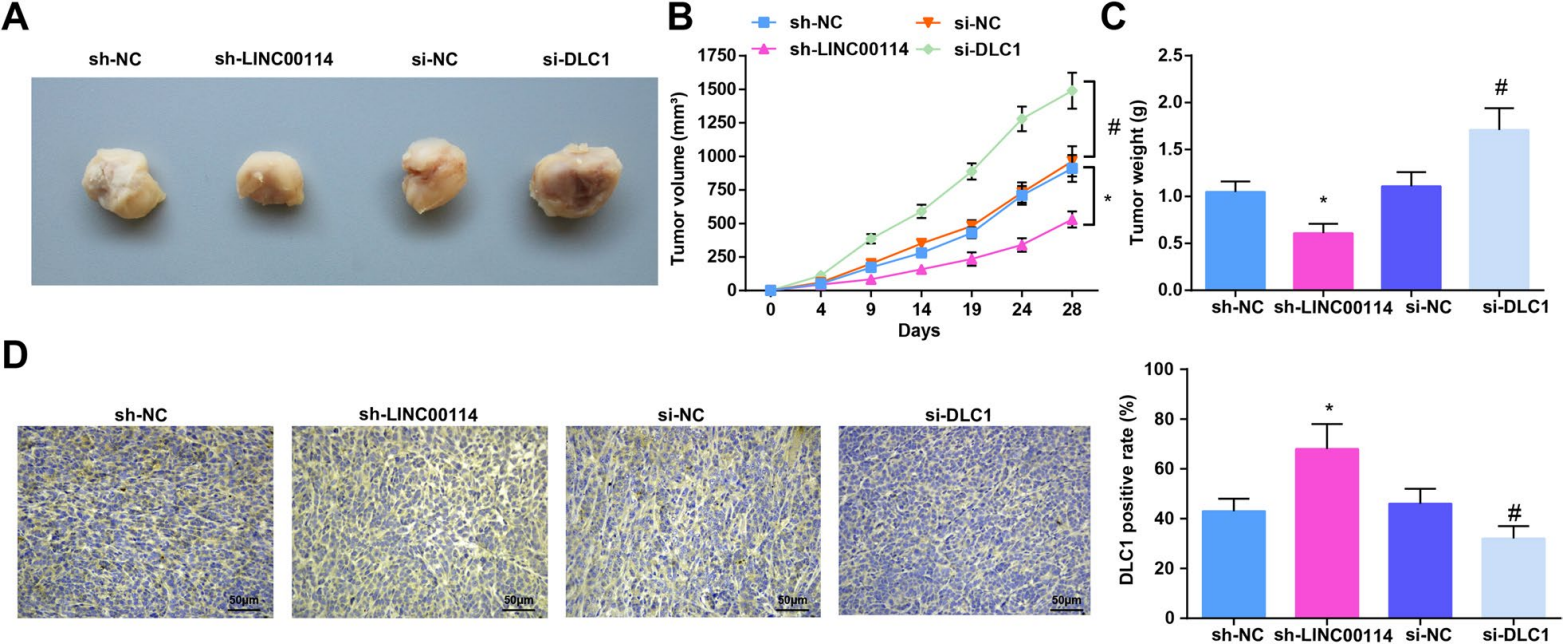
Down-regulating LINC00114 elevates DLC1 expression to delay EC tumor growth in vivo. **A** Final tumor images in nude mice injected with Eca109 stably transfected with sh-LINC00114 or si-DLC1; **B** tumor growth curve of nude mice injected with Eca109 stably transfected with sh-LINC00114 or si-DLC1; **C** tumor weight of nude mice injected with Eca109 stably transfected with sh-LINC00114 or si-DLC1; **D** immunohistochemical detection of DLC1 expression in tumor tissue. Data were shown as mean ± standard deviation. *n* = 5, *P < 0.05 versus the sh-NC group; # *P* < 0.05 versus the si-NC group

Finally, in vivo experiment was performed to further study the role of LINC00114 and DLC1 in EC in vivo. Eca109 cells transfected with sh-LINC00114 or si-DLC1 were injected into BALB/c nude mice (*n* = 5/group). Tumor formation in nude mice implicated that sh-LINC00114 reduced the tumorigenic ability of Eca109 cells while si-DLC1 had the opposite effect in nude mice (Fig. 6A–C). In addition, immunohistochemical detection manifested that sh-LINC00114 raised while si-DLC1 suppressed DLC1 expression in tumors (Fig. 6D).

Collectively, interfering with LINC00114 elevated DLC1 expression to inhibit EC tumor growth in vivo.

## Discussion

EC is a highly aggressive and fatal tumor, causing a great number of deaths annually [34]. ncRNAs, including lncRNAs, are potent biomarkers and therapeutic targets for EC [35]. Despite a sufficient supply of oxygen, tumor cells still need to consume a large amount of glucose to produce lactic acid. This phenomenon is called aerobic glycolysis, or the Warburg effect, which is a unique metabolic mechanism that provides energy to cancer cells. Tumor cells rely on glycolysis, which is a primitive metabolic pathway that can easily be used by cancer cells to obtain energy for growth and spread [27]. Based on that, we specially explored the role of LINC00114 in EC and eventually disclosed that LINC00114 stimulated the biological functions, as well as glycolysis of EC cells via inducing EZH2-mediated H3K27m3 of DLC1.

We found that LINC00114 was up-regulated in both EC tissues and cells, and silence of LINC00114 repressed proliferation, migration, invasion and glycolysis while induced apoptosis of EC cells in vitro, as well as resulted in suppression of tumor formation in vivo. LINC00114 expression is measured to be overexpressed in NPC, and down-regulation of LINC00114 has the inhibitory effects on the proliferative and migratory capacities of cells, as well as re-sensitizes malignant cells to radiotherapy [11]. In the process of CRC, LINC00114 expression trends to up-regulate, and down-regulating LINC00114 inhibits proliferation in vitro, and retards tumor formation in vivo via reducing EZH2/DNA methyltransferase 1-induced methylation of miR-133b promoter [12]. Overall, LINC00114 is a promoter for cancer development, and suppressing LINC00114 may serve to inhibit tumorigenesis, which may be related to DNA methylation. The effect of LINC00114 on glycolysis of EC cells has seldomly been reported, but there are studies highlighting the functions of lncRNAs on glycolysis. For example, We et al. have found that PTPRG-AS1 silencing suppresses the proliferation, migration, as well as glycolysis of ESCC cells [36]. Meanwhile, another study has pointed out that down-regulation of LINC00184 hinders the proliferation, migration, and glycolysis of EC cells [37]. The aforesaid articles are in line with the findings of our study, which needs further confirmation.

Afterward, EZH2 was examined to interact with LINC00114, thus we further studied the role of EZH2 in EC. The outcomes manifested that EZH2 was highly expressed in EC, and functionally reducing EZH2 levels in EC cells weakened cell progression and glycolysis. There is a publication having revealed that EZH2 is up-regulated in patients with EC [14], and depletion of EZH2 has the capacity to block proliferation and migration of EC cells [38]. Besides, EC cells stably expressing EZH2 have stronger ability to migrate and invade whereas down-regulated EZH2 alleviates the ability [39]. Experimentally, EZH2 expression is elevated in ESCC, and ectopic EZH2 could strengthen migration and invasion while suppressing apoptosis of cells [40]. Additionally, inhibition of EZH2 has been demonstrated to retard cell growth and glycolysis in prostate cancer [41]. Moreover, EZH2-mediated H3K27me3 has been explored in cancers. For instance, reduction in EZH2 and H3K27me3 enrichment in the promoter of programmed cell death 4 is associated with impairments in cell proliferation and tumor growth [42]. Also, update research has highlighted that EZH2-mediated H3K27m3 blockade of cyclin-dependent kinase inhibitor 1C could suppress glycolysis, proliferation and migration of pancreatic cancer cells [43]. Commonly, EZH2 serves actively in stimulating tumorigenesis, and EZH2-mediated H3K27m3 might be involved in cancer progression.

Finally, we tested that DLC1 expression was suppressed in response to H3K27m3 enrichment in the promoter of DLC1. In fact, the phenomenon has been detected previously [19]. After that, we navigated that knockdown of DLC1 enhanced growth and glycolysis of EC cells. Accordingly, the function of DLC1 has been revealed, as reflected by the fact that elevating DLC1 attenuates the aggressive activities of hepatocellular carcinoma cells [44]. Furthermore, it has been declared that if reducing EZH2 expression in triple-negative breast cancer cells, DLC1 expression is elevated and curcumin-induced inhibition of cancer growth is facilitated [45]. In short, DLC1 deficiency reduces an opportunity for cancers to progress. As for the role of DLC1 in glycolysis of EC cells, our study suggested that knockdown of DLC1 enhanced glycolysis of EC cells, which was our new finding and should be verified in our future research.

Generally, our research figures out that silence of LINC00114 slows down EC progression through up-regulating EZH2-mediated DLC1. It is the first time to probe the integral actions of LINC00114, EZH2 and DLC1 in EC, renewing the molecular mechanism underlying EC. A more thorough insight into the mechanism of EC still depends on a great amount of researches.

## Supplementary Information

**Additional file 1: Table S1.** Primers used in our study.

**Additional file 2: Fig. S1.** Suppression of LINC00114 blunts invasion and migration of EC cells. **A** Colony formation assay measured the colony forming ability of EC cells after LINC00114 interference; **B** transwell assay detected EC cell migration after LINC00114 interference; **C** transwell assay detected EC cell invasion after LINC00114 interference; **D** flow cytometry detected cell apoptosis after LINC00114 interference; data were shown as mean ± standard deviation and evaluated by ANOVA and ANOVA and Tukey method. *N* = 3, *P < 0.05 vs. the sh-NC group.

**Additional file 3: Fig. S2.** Silencing EZH2 inhibits invasion and migration of EC cells. **A** Colony formation assay measured the colony forming ability of EC cells after EZH2 interference; **B** Transwell assay detected EC cell migration after EZH2 interference; **C** transwell assay detected EC cell invasion after EZH2 interference; **D** flow cytometry detected cell apoptosis after EZH2 interference; data were shown as mean ± standard deviation and evaluated by ANOVA and ANOVA and Tukey method. *N* = 3; *P < 0.05 vs. the sh-NC group.

**Additional file 4: Fig. S3.** Knocking down DLC1 enhances invasion and migration of EC cells. **A** Colony formation assay measured the colony forming ability of EC cells after DLC1 interference; **B** transwell assay detected EC cell migration after DLC1 interference; **C** transwell assay detected EC cell invasion after DLC1 interference; **D** flow cytometry detected cell apoptosis after DLC1 interference; data were shown as mean ± standard deviation and evaluated by ANOVA and ANOVA and Tukey method. *N* = 3; * *P* < 0.05 vs. the si-NC group.

**Additional file 5: Fig. S4.** Knocked down DLC1 mitigates the role of suppressed LINC00114 in colony formation ability and apoptosis of EC cells. **A** Flow cytometry detected cell apoptosis after co-transfection with sh-LINC00114 and si-DLC1; **B** colony formation assay measured the colony forming ability of EC cells after co-transfection with sh-LINC00114 and si-DLC1; data were shown as mean ± standard deviation and evaluated by ANOVA and ANOVA and Tukey method. *N* = 3; *P < 0.05 vs. the si-NC group. # *P* < 0.05 vs. the sh-LINC00114 + si-NC group.

**Additional file 6: Fig. S5.** Knocked down DLC1 mitigates the role of suppressed LINC00114 in invasion and migration of EC cells. **A** Transwell assay detected EC cell migration after co-transfection with sh-LINC00114 and si-DLC1; **B** transwell assay detected EC cell invasion after co-transfection with sh-LINC00114 and si-DLC1; data were shown as mean ± standard deviation and evaluated by ANOVA and ANOVA and Tukey method. *N* = 3; *P < 0.05 vs. the si-NC group. # *P* < 0.05 vs. the sh-LINC00114 + si-NC group.

## Abbreviations

EC: Esophageal cancer
EZH2/DLC1: Enhancer of zeste homolog 2/deleted in liver cancer 1
ESCC: Esophageal squamous cell carcinoma
LncRNA: Long non-coding RNA
LINC00114: Long non-coding RNA 00114
NPC: Nasopharyngeal carcinoma
CRC: Colorectal cancer
EZH2: Enhancer of zeste homolog 2
DLC1: Deleted in liver cancer 1
HEEC: Human esophageal epithelial cells
FBS: Fetal bovine serum
DMEM: Dulbecco’s modified Eagle's medium
RT-qPCR: Reverse transcription quantitative polymerase chain reaction
HRP: Horseradish peroxidase
GAPDH: Glyceraldehyde-3-phosphate dehydrogenase
ATP: Adenosine triphosphate
CCK: Cell counting kit
RIP: RNA immunoprecipitation
ChIP: Chromatin immunoprecipitation
PI: Propidium iodide
FITC: Fluorescein isothiocyanate
