# Supplementary material for: LINC00114 stimulates growth and glycolysis of esophageal cancer cells by recruiting EZH2 to enhance H3K27me3 of DLC1
Source: Clin Epigenetics. 2022 Apr 12;14:51. doi: 10.1186/s13148-022-01258-y (PMC9006613; doi:10.1186/s13148-022-01258-y)
Supplement: Supplementary file 1 — Additional file 1: Table S1. Primers used in our study. [file 13148_2022_1258_MOESM1_ESM.docx]

**Supplementary Table 1** Primers used in our study

| Primers | Forward (5’-3’) | Reverse (5’-3’) |
| --- | --- | --- |
| LINC00114 | CAAGAGGAAGGTGGGAGCTG | ACCCAGGTGATGAGGAGGAA |
| EZH2 | AATCAGAGTACATGCGACTGAGA | GCTGTATCCTTCGCTGTTTCC |
| DLC1 | GGCGGAGTACCGAGGCAT | GTGATCGCTAAAGGACCTCCG |
| GAPDH | GGGAGCCAAAAGGGTCAT | GAGTCCTTCCACGATACCAA |

Note: LINC00114, long non-coding RNA LINC00114; EZH2, enhancer of zeste homolog 2; DLC1, deleted in liver cancer 1; GAPDH, glyceraldehyde-3-phosphate dehydrogenase.
