# Supplementary figures and images for: LINC00114 stimulates growth and glycolysis of esophageal cancer cells by recruiting EZH2 to enhance H3K27me3 of DLC1
Source: Clin Epigenetics. 2022 Apr 12;14:51. doi: 10.1186/s13148-022-01258-y (PMC9006613; doi:10.1186/s13148-022-01258-y)

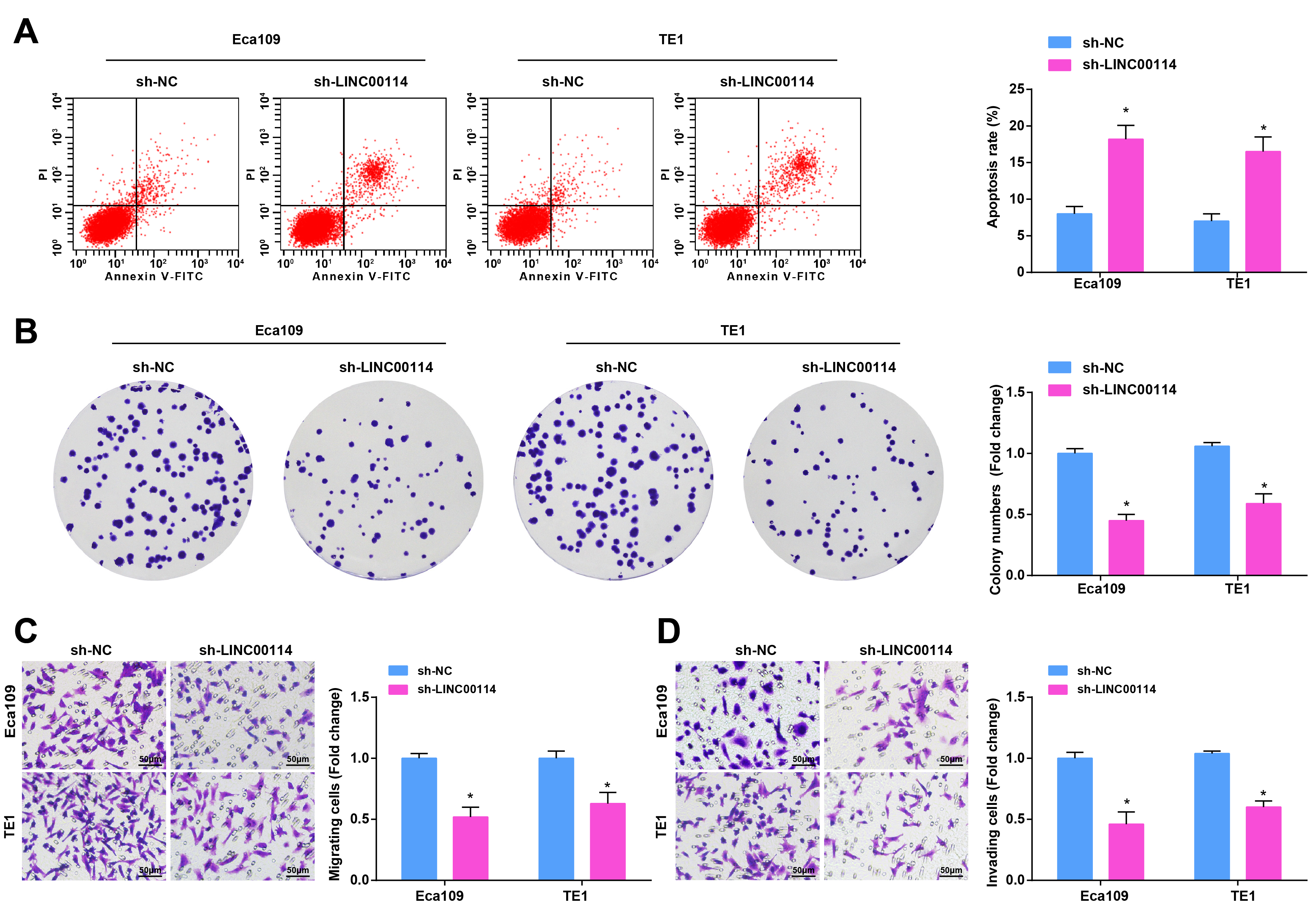

Supplement: Supplementary file 2 — Additional file 2: Fig. S1. Suppression of LINC00114 blunts invasion and migration of EC cells. A Colony formation assay measured the colony forming ability of EC cells after LINC00114 interference; B transwell assay detected EC cell migration after LINC00114 interference; C transwell assay detected EC cell invasion after LINC00114 interference; D flow cytometry detected cell apoptosis after LINC00114 interference; data were shown as mean ± standard deviation and evaluated by ANOVA and ANOVA and Tukey method. N = 3, *P < 0.05 vs. the sh-NC group. [file 13148_2022_1258_MOESM2_ESM.jpg]

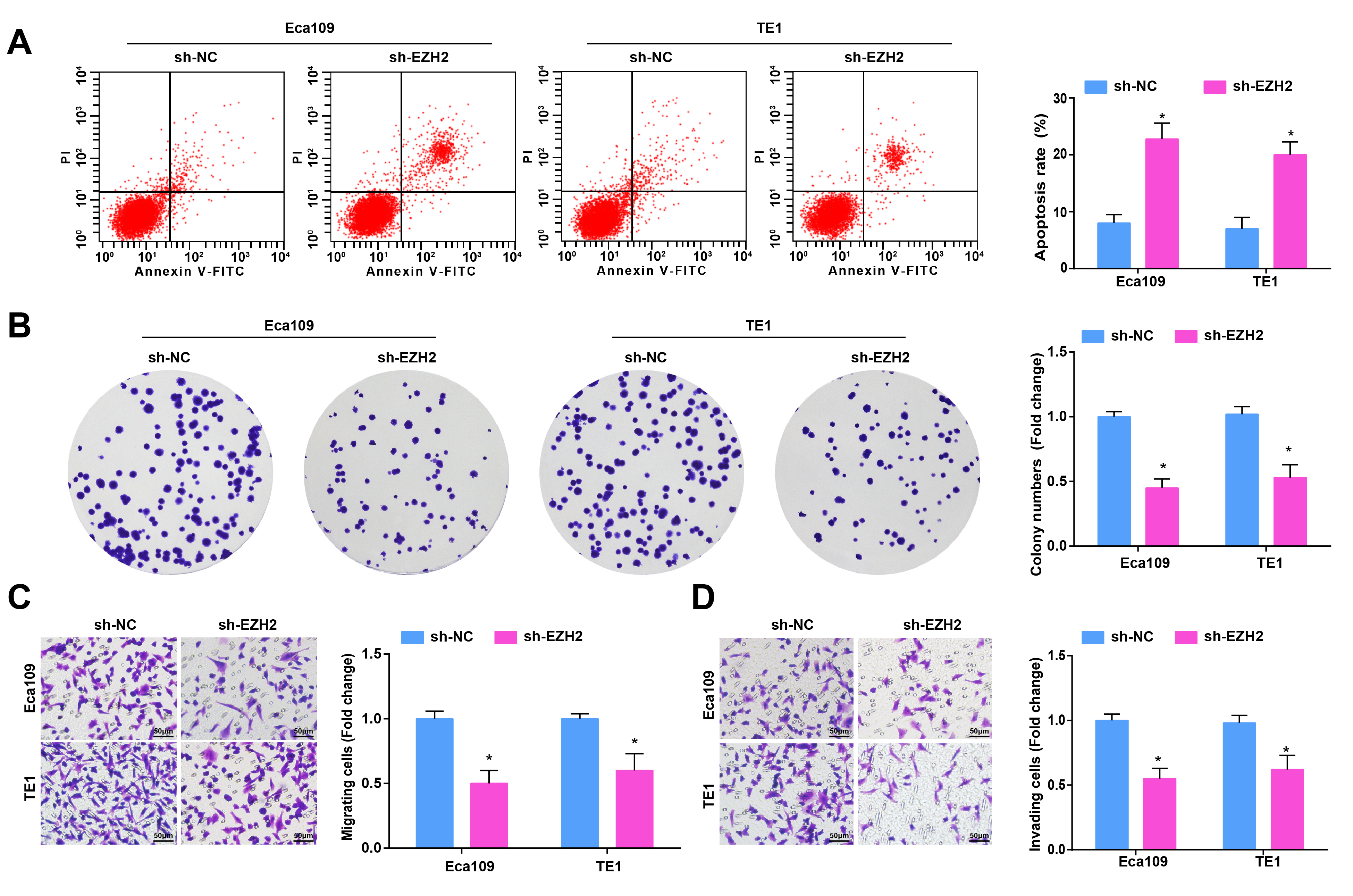

Supplement: Supplementary file 3 — Additional file 3: Fig. S2. Silencing EZH2 inhibits invasion and migration of EC cells. A Colony formation assay measured the colony forming ability of EC cells after EZH2 interference; B Transwell assay detected EC cell migration after EZH2 interference; C transwell assay detected EC cell invasion after EZH2 interference; D flow cytometry detected cell apoptosis after EZH2 interference; data were shown as mean ± standard deviation and evaluated by ANOVA and ANOVA and Tukey method. N = 3; *P < 0.05 vs. the sh-NC group. [file 13148_2022_1258_MOESM3_ESM.jpg]

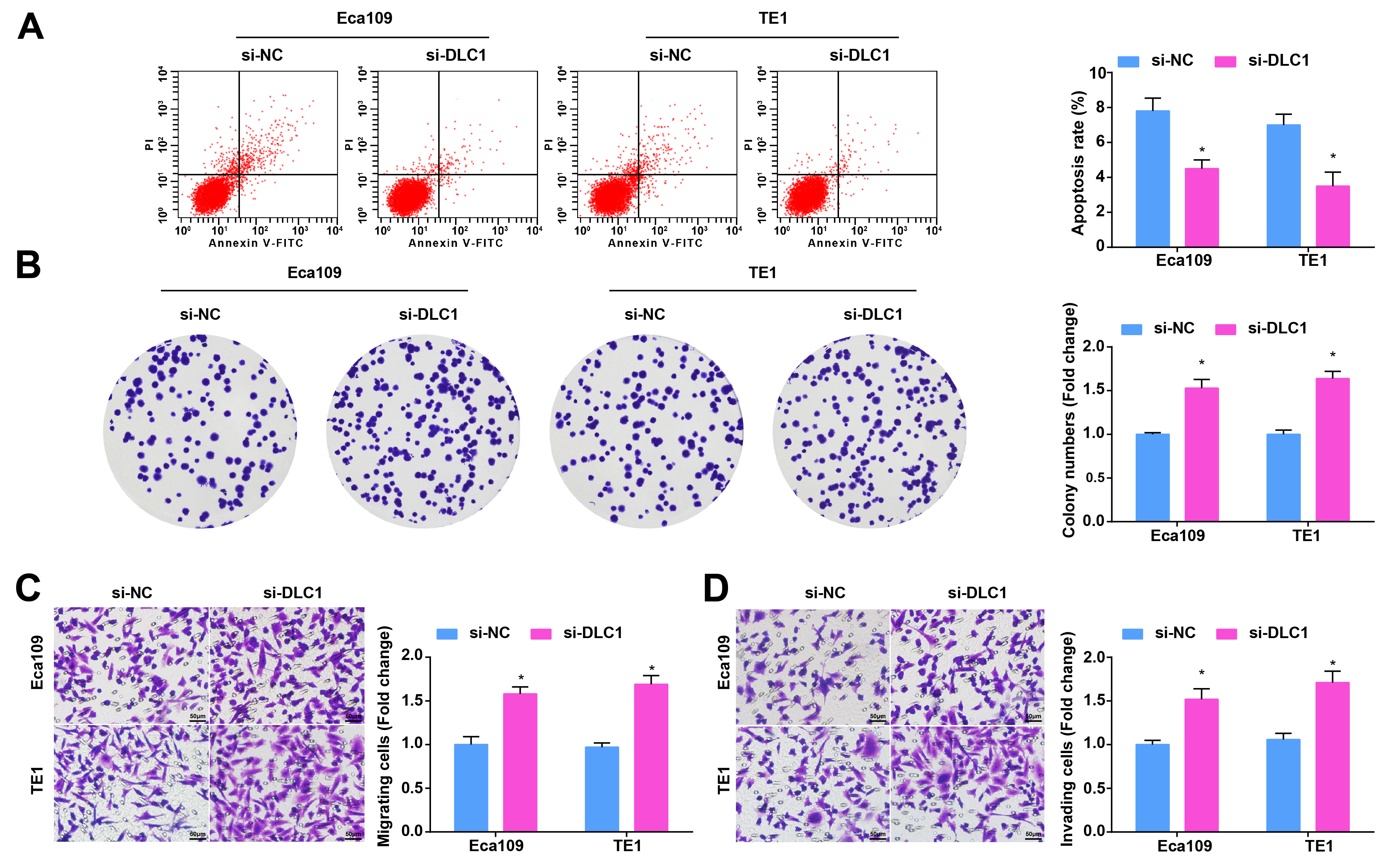

Supplement: Supplementary file 4 — Additional file 4: Fig. S3. Knocking down DLC1 enhances invasion and migration of EC cells. A Colony formation assay measured the colony forming ability of EC cells after DLC1 interference; B transwell assay detected EC cell migration after DLC1 interference; C transwell assay detected EC cell invasion after DLC1 interference; D flow cytometry detected cell apoptosis after DLC1 interference; data were shown as mean ± standard deviation and evaluated by ANOVA and ANOVA and Tukey method. N = 3; * P < 0.05 vs. the si-NC group. [file 13148_2022_1258_MOESM4_ESM.jpg]

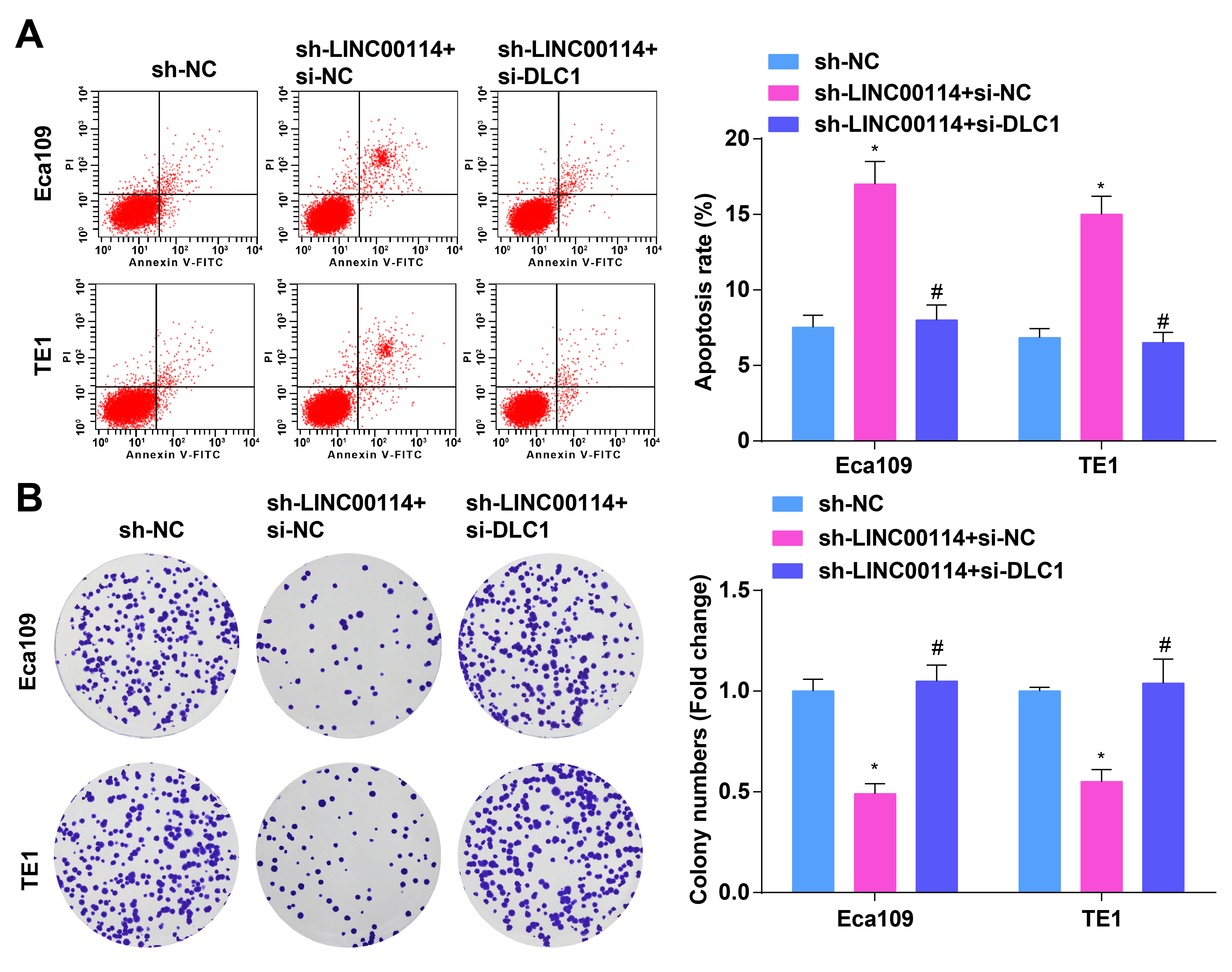

Supplement: Supplementary file 5 — Additional file 5: Fig. S4. Knocked down DLC1 mitigates the role of suppressed LINC00114 in colony formation ability and apoptosis of EC cells. A Flow cytometry detected cell apoptosis after co-transfection with sh-LINC00114 and si-DLC1; B colony formation assay measured the colony forming ability of EC cells after co-transfection with sh-LINC00114 and si-DLC1; data were shown as mean ± standard deviation and evaluated by ANOVA and ANOVA and Tukey method. N = 3; *P < 0.05 vs. the si-NC group. # P < 0.05 vs. the sh-LINC00114 + si-NC group. [file 13148_2022_1258_MOESM5_ESM.jpg]

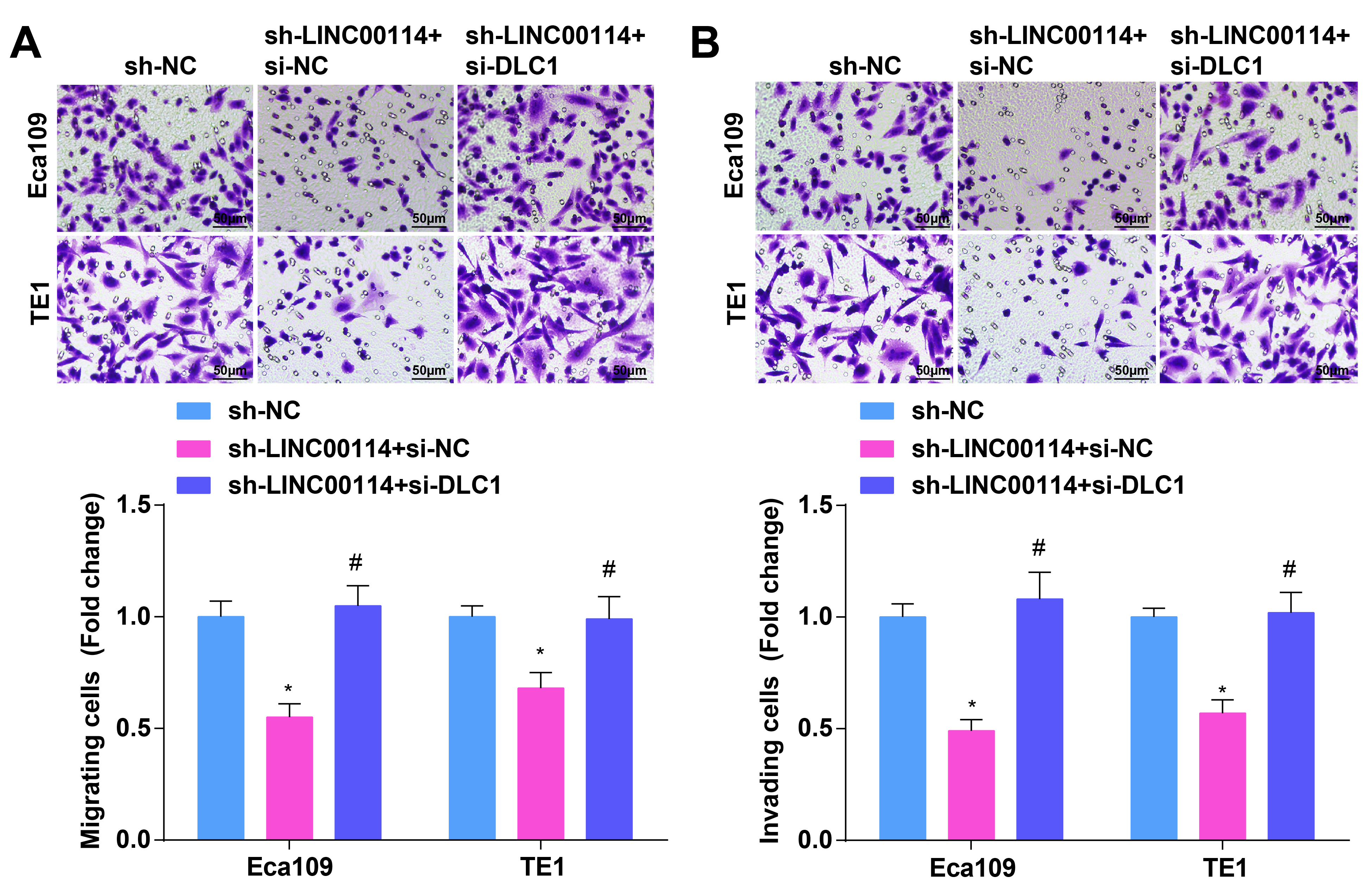

Supplement: Supplementary file 6 — Additional file 6: Fig. S5. Knocked down DLC1 mitigates the role of suppressed LINC00114 in invasion and migration of EC cells. A Transwell assay detected EC cell migration after co-transfection with sh-LINC00114 and si-DLC1; B transwell assay detected EC cell invasion after co-transfection with sh-LINC00114 and si-DLC1; data were shown as mean ± standard deviation and evaluated by ANOVA and ANOVA and Tukey method. N = 3; *P < 0.05 vs. the si-NC group. # P < 0.05 vs. the sh-LINC00114 + si-NC group. [file 13148_2022_1258_MOESM6_ESM.jpg]
